# *In silico* Therapeutics for Neurogenic Hypertension and Vasovagal Syncope

**DOI:** 10.3389/fnins.2015.00520

**Published:** 2016-01-21

**Authors:** Tijana Bojić, Vladimir R. Perović, Sanja Glišić

**Affiliations:** ^1^Laboratory of Radiobiology and Molecular Genetics-080, Institute of Nuclear Sciences Vinča, University of BelgradeBelgrade, Serbia; ^2^Center for Multidisciplinary Research-180, Institute of Nuclear Sciences Vinča, University of BelgradeBelgrade, Serbia

**Keywords:** *in silico* analysis, neurocardiovascular diseases, neurogenic hypertension, protein-protein interaction, vasovagal syncope

## Abstract

Neurocardiovascular diseases (NCVD) are the leading cause of death in the developed world and will remain so till 2020. In these diseases the pathologically changed nervous control of cardiovascular system has the central role. The actual NCV syndromes are neurogenic hypertension, representing the sympathetically mediated disorder, and vasovagal syncope, which is the vagally mediated disorders. Vasovagal syncope, the disease far from its etiological treatment, could benefit from recruiting and application of antimuscarinic drugs used in other parasympathetic disorders. The informational spectrum method (ISM), a method widely applied for the characterization of protein-protein interactions in the field of immunology, endocrinology and anti HIV drug discovery, was applied for the first time in the analysis of neurogenic hypertension and vasovagal syncope therapeutic targets. *In silico* analysis revealed the potential involvement of apelin in neurogenic hypertension. Applying the EIIP/ISM bioinformatics concept in investigation of drugs for treatment of vasovagal syncope suggests that 78% of tested antimuscarinic drugs could have anti vasovagal syncope effect. The presented results confirm that ISM is a promissing method for investigation of molecular mechanisms underlying pathophysiological proceses of NCV syndromes and discovery of therapeutics targets for their treatment.

## Introduction

NCVD are the syndromes where autonomic nervous system (Zoccoli et al., 2001; Bojic, 2003) dysfunction plays a dominant etiological role (Goldstain, 2001; Bojic et al., 2012a,b). NCV disorders can be classified as sympathetically mediated disorders (i.e., neurogenic hypertension, NH) vs. vagally mediated disorders (i.e., vasovagal syncope, VVS), though in many disorders both systems are dysfunctional (Goldstain, 2001).

NH is characterized by an increased level of sympathetic nervous activity (SNA) (Fisher and Paton, 2011). SNA is in reciprocal interaction with the number of important systems for the pathophysiological profile of NH, like inflammation, angiotension II system and vascular dysfunction. Recently, due to its potential causal role in the genesis of NH, an emphasis was put on the role of oxidative stress in brain stem structures.

Reactive oxygen spices (ROS) in the brain can be generated in angiotesin II dependent and angiotensin II independent manners. The most studied mechanism for investigating the potential causal treatment of NH is Ang 1-7-MAS receptor-NO mechanism (Zimmerman, 2011). This mechanism counteracts prohypertensive actions of ROS and represents a good choice for investigation of therapeutic candidate by Informational Spectrum Method (ISM).

In the case of VVS, Nucleus Tractus Solitarii (NTS) in the brain stem is stimulated either directly (central VVS) or indirectly (peripheral VVS), provoking an enhancement of vagal tone and withdrawal of SNA tone. This dual response causes a continuum of cardiovascular phenotype responses. At the “vagal end” it leads to the cardioinhibitory type of VVS (bradicardia as a dominant cause of the VVS), while on the “sympathetic end” causes the vasodepressor type of VVS (hypotension as a dominant cause of the VVS). Mixed type of VVS is on the midway between these two extremes.

The treatment of VVS involves a layered approach with a combination of lifestyle changes, physical maneuvers, medications, and implantable devices. The vast majority of patients with VVS can be adequately controlled with non-pharmacological approaches and do not require pharmacological treatment (Raj and Coffin, 2013). There is, however, the minority of patients with refractory and recurrent VVS who can benefit from effective pharmacotherapy.

Since the majority of the VVS patients suffer from mixed and cardioinhibitory type of VVS, we took under consideration the modulation of vagal tone as the *in silico* strategy for the new anti VVS drugs.

The therapy of NH and VVS is far from efficient etiological treatment. The development of new drugs for treatment of NH and VVS is time and money consuming process, which can be accelerated by *in silico* screening of the molecular libraries for candidate novel drugs and by repurposing of approved drugs.

The aim of this study was to apply EIIP/ISM approach to identify candidate neuropeptides and small-molecules for the potential therapeutics of NH and VVS. The study may lead to new insights in the field of neurocardiovascular pharmacotherapy and pathophysiology.

## Methods

### The long range molecular interactions

Current concepts of intermolecular interactions in biological systems are based on the surface complementarity between interacting biomolecules and assumption that the first contact between interacting molecules is achieved accidentally by the thermal motions that cause molecular wander. If proteins are considered as spheres of 18 Å radius (typical of a small protein), and if spheres associate with every contact, without regard to orientation, the diffusion-limited association rate constant, calculated according to Smoluchowski's equation (Smoluchowski, 1916) is 7 × 10^9^ M^−1^s^−1^. However, before chemical bond formation takes place, reacting molecular regions must be positioned close enough (at a distance of ~2 Å) and the appropriate atoms must be held in the correct orientation for the reaction that is to follow, because the attractive forces involved in the recognition and binding of molecules include all the weak non-covalent forces. It means that the protein's binding site is only a small fraction (~0.1%) of the surface area. Taking into account this limitation, the diffusion-limited association rate constant, predicted from a three-dimensional (3D) “random diffusion” model and calculated according Smoluchowski's equation is ~10^6^ M^−1^s^−1^ for a protein-ligand and ~10^3^ M^−1^s^−1^ for a protein-protein interaction. Northrup and Erickson have noted that protein-protein association generally occurs at rates that are 10^3^–10^4^ times faster than would be expected from simple considerations of collision frequencies and strict orientation effects which assume that productive binding occurs only when the molecules collide within 2 Å of their final binding site (Northrup and Erickson, 1992).

### Electron-ion interaction potential (EIIP)

In order to overcome the discrepancy between theoretically estimated values and real values of the associated rate constant for a intermolecular interactions in biological systems, the long-range intermolecular interactions (distances between 5 and 1000 A) between interacting molecules was proposed (Veljkovic, 1980). It has been showed that the EIIP and the average quasivalence numbers (AQVN) Z^*^ represent essential molecular descriptors which determines the long-range properties of biological molecules (Veljkovic, 1980). These two molecular descriptors are defined by the following equations:

(1) $${\text{Z}}^{*}=\overset{\text{m}}{\sum }{\text{n}}_{\text{i}}{\text{Z}}_{\text{i}}\text{/N}$$

Where:

i, Type of the chemical element;

Z, Valence of the ith chemical element;

n, Number of the ith chemical element atoms in the compound;

m, Number of types of chemical elements in the compond;

N, total number of atoms.

(2) $$\begin{array}{r} \text{EIIP}=0.25{\text{Z}}^{*}\text{sin}\left(1.04\text{π}{\text{Z}}^{*}\right)∕2\text{π} \end{array}$$

The EIIP values calculated according to the Equation (2) are in Rydbergs (Ry = 13.6 eV).

### Informational spectrum method (ISM)

The ISM a virtual spectroscopy method for calculation of the long-range properties of biological macromolecules, is based on a model that assigns to each amino acid a defined parameter describing a physico-chemical property involved in the biological activity of the protein and corresponding to electron-ion interaction potential (EIIP) (Veljkovic et al., 1985).

ISM method consists in three basic steps:

1. Transformation of alphabetic code of primary protein structure into a sequence of numbers representing EIIP of each component.
2. Conversion of numerical sequence by fast Fourier Transformation into information spectrum, which reveals dominant freqency peaks of the whole organic molecule.
3. Consensus Information Spectrum (CIS) analysis between information spectrums of two potentially similar or interactive molecules, which reveales functional locus of the interaction of two molecules.

Peak frequencies in CIS are common frequency components for the analyzed sequences. A measure of similarity for each peak is the signal-to-noise ratio (S/N), the ratio between the signal intensity at one particular IS frequency and the main value of the whole spectrum.

**Figure 1:**
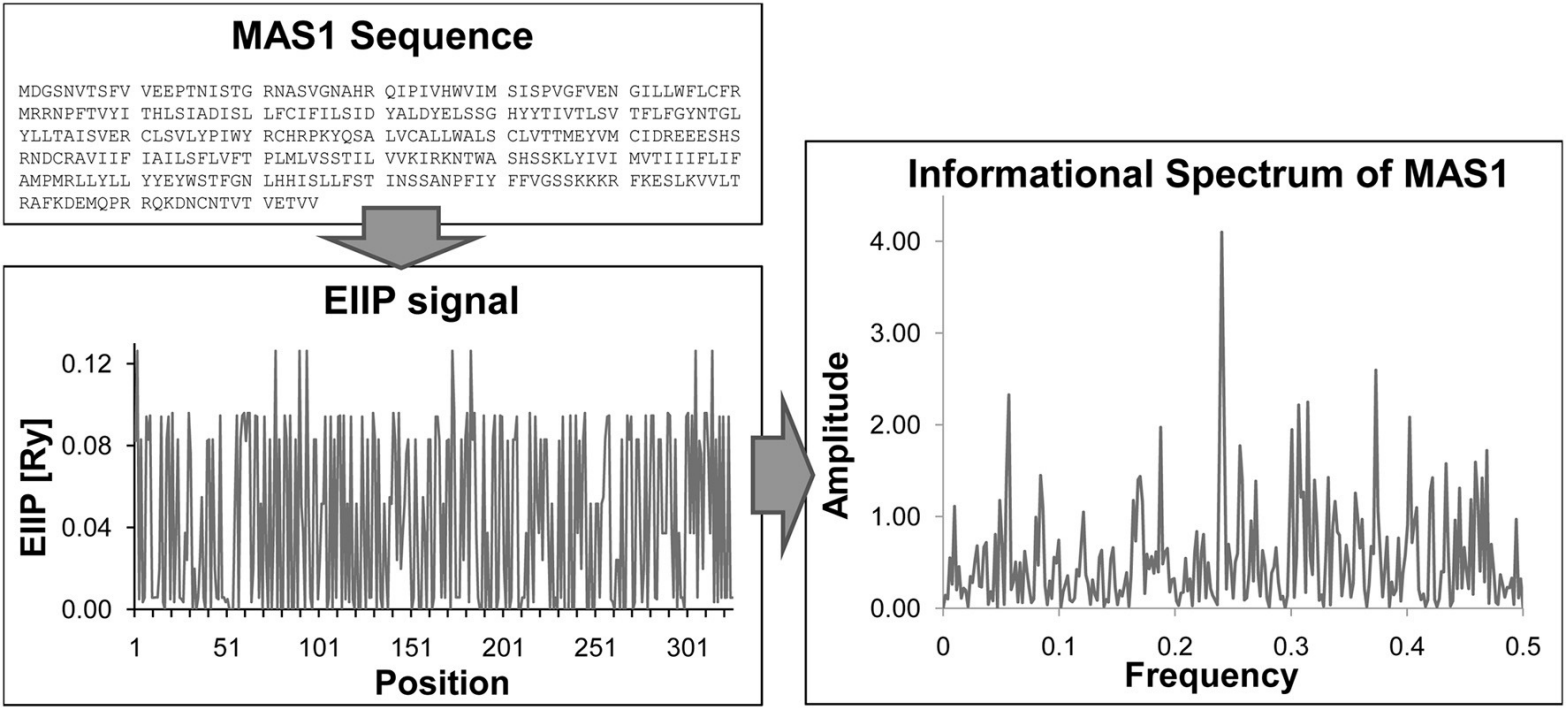
**Basic steps of ISM method**. Sequence → Transformation of primary protein sequence into sequence of numbers, by assigning of EIIP to each amino acid → Numerical presentation → Discrete Fourier transformation → Informational spectrum (IS).

Schematic Presentation of ISM is given in Figure 1.

The strong connection between EIIP and Z^*^ molecular descriptors of small molecules and their biological activities (carcinogenisity, antibiotic activity, antiviral activity, toxicity, etc.) has been documented (for review see Veljkovic et al., 2011 and references therein).

The method has been successful in identification of functional protein domains representing candidate therapeutic targets for anti-HIV drugs (Veljkovic et al., 2007), anthrax (Doliana et al., 2008), and human influenza viruses (Veljkovic et al., 2009a,b; Perovic et al., 2013). It is recognized and used in more than 100 research centers worldwide (Veljkovic et al., 2011).

### Sequences and molecular formulas of compounds

Sequences were retrieved from UniProt Database with following accession numbers: (a) sequences of primates MAS1: Homo sapiens P04201, Pan troglodytes H2QU00, Macaca mulatta F7GJU7, Pongo abelii H2PL76GN, Chlorocebus sabaeus A0A0D9RJ10, Gorilla gorilla gorilla G3R4L5, Papio anubis A0A096NZT0. (b) sequences of primates Angiotensin 1-7: Homo sapiens P01019, Pan troglodytes H2Q1B7, Macaca mulatta G7MFR4, Gorilla gorilla gorilla Q9GLP6. (C) sequences of primates Apelin: Homo sapiens Q9ULZ1, Macaca mulatta F7GX01, Gorilla gorilla gorilla G3S9L8, Chlorocebus sabaeus A0A0D9R7Q2. Sequences are shown in Data Sheet 1.

Molecular formulas of compounds: muscarinic modulators from US Patent 7786308, US Patent 7378447US, and Patent Application 20100311746A1.

## Results

**Figure 2:**
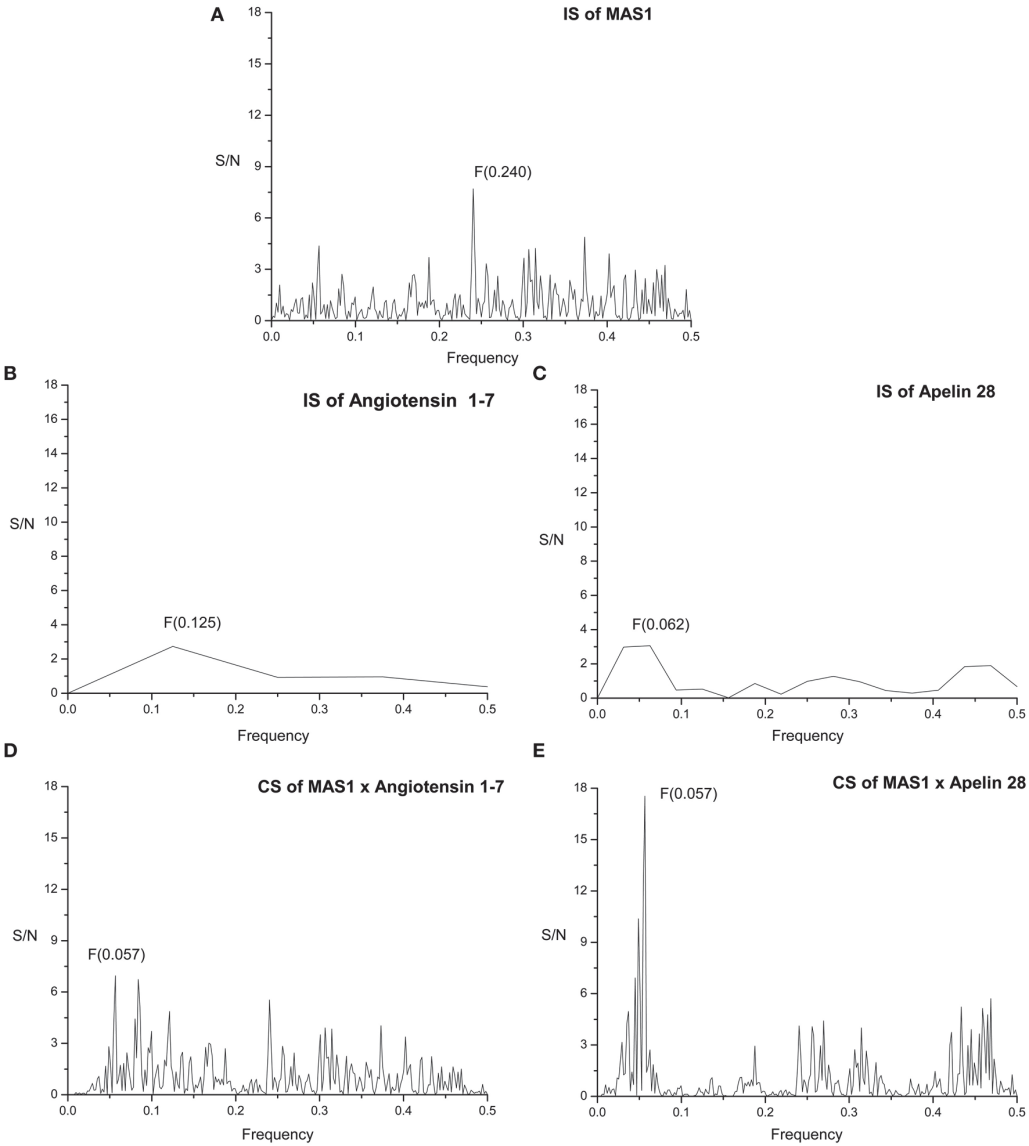
**Informational spectrum (IS) of (A) MAS1, (B) angiotensin 1-7, (C) apelin 28, and consensus informational-spectrum (CIS) of (D) MAS1 and angiotensin 1-7 and (E) MAS1 and apelin**.

The primary structure of proteins encodes the information represented by the informational spectrum (IS) frequencies that correspond to the protein biological function. Mutually interacting proteins share common information represented by peaks in their cross-spectrum (Veljkovic et al., 2008). IS of human MAS receptor (MAS1) is presented on Figure 2A. It contains characteristic peak at the frequency *F*_(0.240)_.

**Figure 3:**
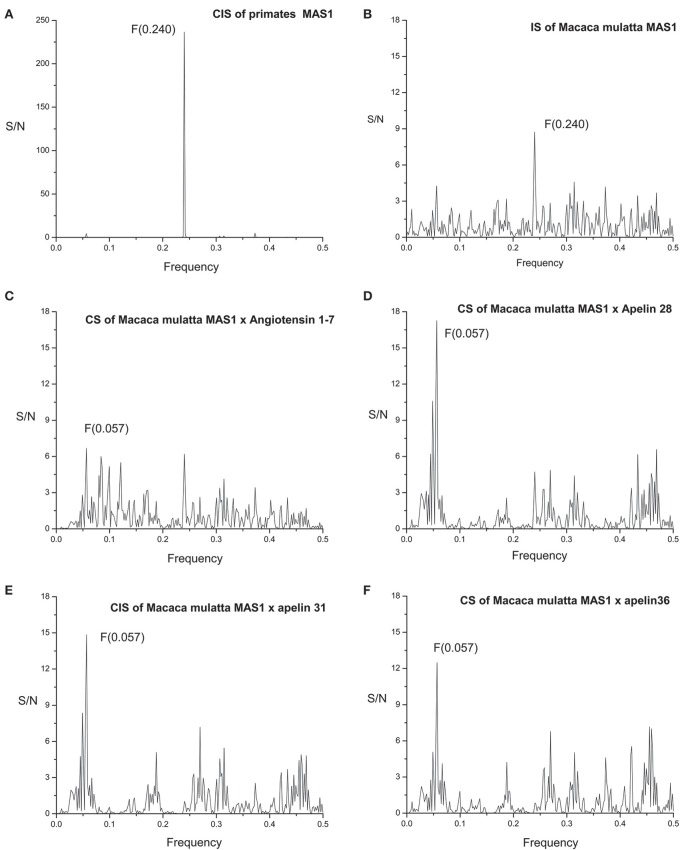
**(A)** Consensus informational spectrum (CIS) of primates MAS1; **(B)** Informational spectrum (IS) of MAS1 of Macaca mulatta; **(C)** CS of Macaca mulatta MAS1 and angiotensin 1-7 and **(D)** CS of Macaca mulatta MAS1 and apelin 28, **(E)** CS of Macaca mulatta MAS1 and apelin 31 **(F)** CS of Macaca mulatta MAS1 and apelin 36.

MAS1 sequences are highly homologous among primates [see Supplementary Material: Data Sheet 1 and Exported Multiple Sequence Alignment (Edgar, 2004) of MAS1 as artwork using Jalview (Waterhouse et al., 2009; Image 1)]. Informational spectrum (IS) of MAS1 of Macaca mulatta (Figure 3B) and consensus informational spectrum (CIS) of MAS1 of primates are shown in Figure 3A. It can be seen that information represented by the CIS frequency *F*_(0.240)_, is evolutionary conserved among proteins having the same biological function, as it was shown previously (Glisic et al., 2008).

The sequences of peptides Angiotensin 1-7 and Apelins of other primates are the same as the sequences of human peptides (Data Sheet 1) and consequently spectra are the same.

Figure 2B represents IS of the of human Angiotensin 1-7 (Ang 1-7). By performing cross-spectral analysis (CS) of MAS receptor and Ang 1-7 we have identified that these two molecules share common information corresponding to the IS frequency *F*_(0.057)_ (Figure 2D). To confirm the reliability of our findings we have performed CS analysis of Macaca mulatta MAS1 and Ang 1-7 and identified the same frequency *F*_(0.057)_ as dominant and evolutionary conserved (Figure 3C).

We further apply ISM analyses to identify new peptide interactors of human MAS1 representing potential candidate therapeutic agents. To identify peptides which share the common information represented by the frequency component *F*_(0.057)_, 270 human peptides of the Human Neuropeptide sequence database (Kim et al., 2011) were screened by ISM and peptide apelin 28 (Figure 2C) was found to be among the peptides with the highest amplitudes and Signal/Noise values at the frequency *F*_(0.057)_ in CIS with MAS1 (Figure 2E). Similar results were obtained for apelin-31 and apelin-36. In further analysis it was shown that the information represented by CS frequency for Macaca mulatta MAS receptor and apelins is the same (Figures 3D–F). According to the IS criterion these peptides are the potential candidate interactors of MAS1. Presented results indicate apelin as potential modulator of MAS1 receptor and novel candidate for treatment of NH.

**Figure 4:**
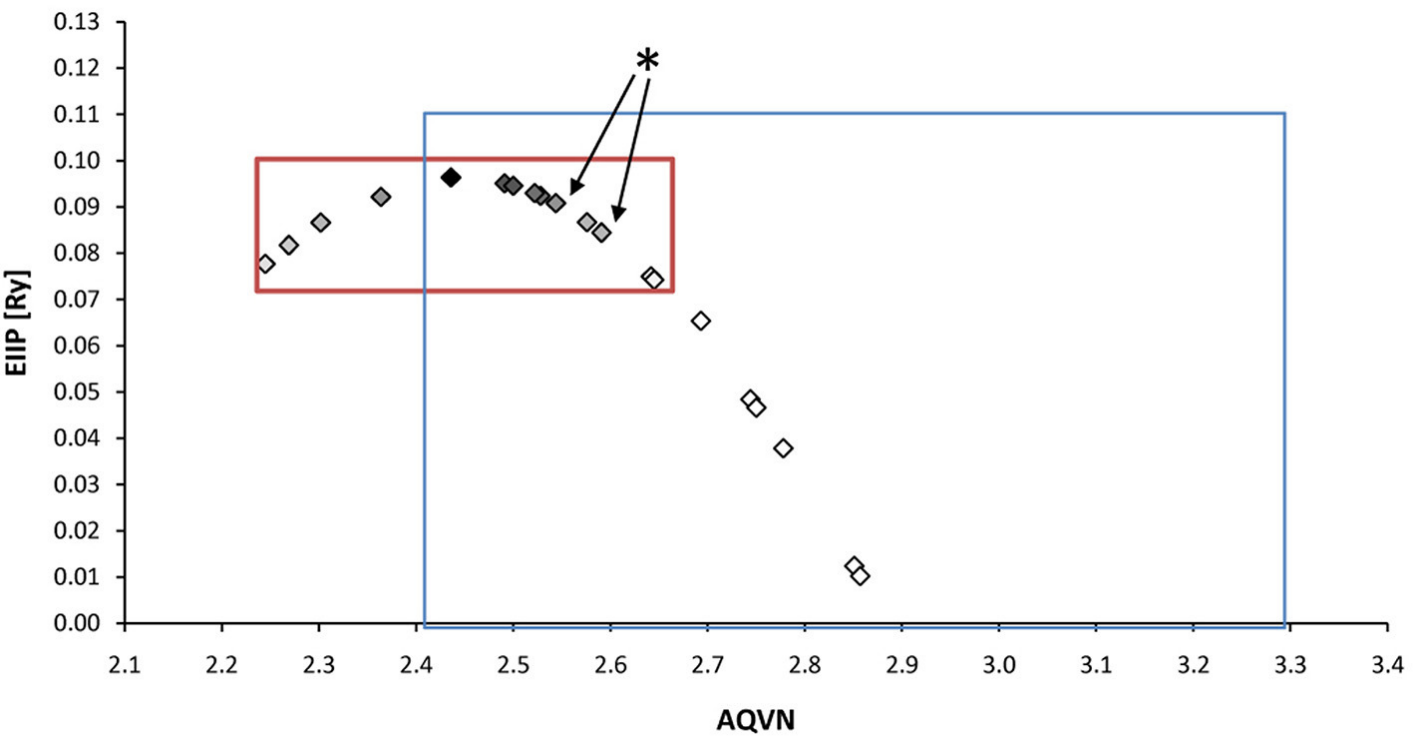
**Schematic presentation of the EIIP/AQVN criterion for selection of candidate antimuscarinic drugs with potential therapeutic effect on VVS**. Active domain (red): AQVN (2.25–2.65), EIIP (0.074–0.096). Chemical space (blue) AQVN (2.40–3.30) EIIP (0.000–0.116) EIIP/AQVN domain of homologous distribution of >90% compounds from PubChem Compound Database. Statistics: inside the active domain: 21 (78%), outside the active domain: 6 (22%). Asterisk (^*^)-position of atropine and propentheline bromide, known anti VVS drugs, within AQVN/EIIP active domain.

**Figure 5:**
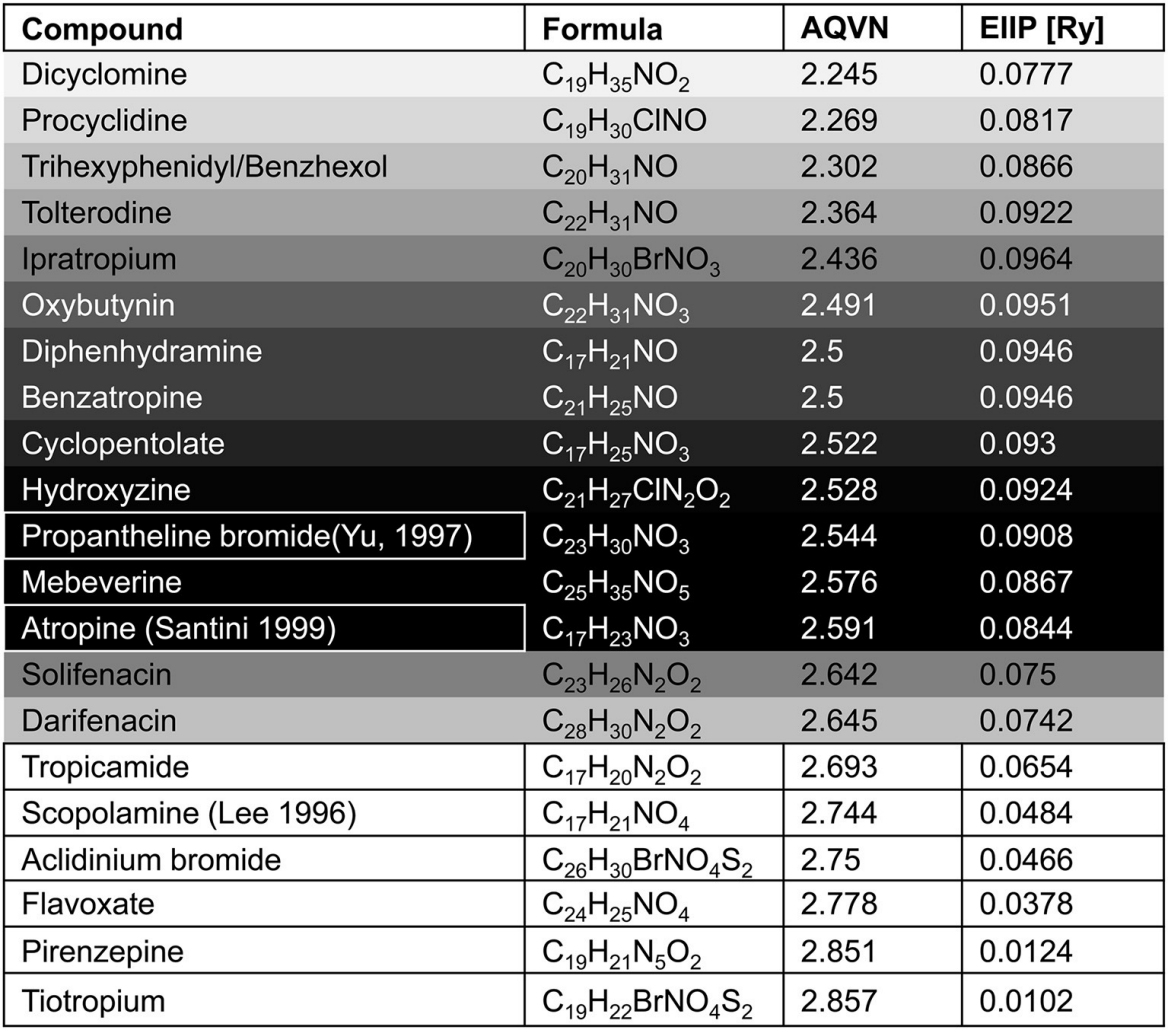
**The list of antimuscarinic drugs tested for their AQVN/EIIP**. Shaded fields- antimuscarinic drugs within the active domain (Figure 3), white fields-antimuscarinic drugs out of the active domain, bordered fields- antimuscarinic drugs tested for their therapeutical effect on VVS. Probability for the anti VVS activity of muscarinicdrugs, assessed by EIIP/AQVN criterion, is represented with the gray scale (black-the highest probability, white-the lowest probability).

With respect to the muscarinic antagonists applied in the therapy of VVS (atropine, propanteline bromide, and scopolamine), on the basis of AQVN/EIIP analysis we analyzed the distribution of other muscarinic antagonists which are not tested for their efficacy in the treatment of VVS (Figures 4, 5). The analysis revealed that 78% of tested drugs are in the active AQVN/EIIP domain, implying that there is consistent number of known antimuscarinic drugs that might have a therapeutic impact on VVS.

We also analyzed muscarinic receptor modulators from three patents which are randomly selected among more than 30,000 patents from the patent database (http://www.freepatentsonline.com), in order test the suitability of the proposed EIIP/AQVN filter. Results of this analysis are presented in Table 1. As can be seen, all patented compounds are located within the active EIIP/AQVN domain (Figure 4).

**Table 1:** **EIIP/AQVN values of patented muscarinic modulators: Muscarinic modulators (US Patent 7786308), Muscarinic agonists (US Patent 7378447), and Modulators of muscarinic receptors (US Patent Application 20100311746A1)**.

**EIIP [RY]**	**AQVN**	**Chemical formula**	**Compound**
**Patent title: Muscarinic modulators (US Patent 7786308**			
0.0845	2.590	C23H31FN2O4	ethyl 3-(4-(4-fluorophenyl)(methoxycarbonyl) amino)piperidin-1-yl)-8-azabicyclo[3.2.1]octane-8-carboxylate
0.0886	2.562	C23H33FN4O3	3-{4-[1-(4-Fluoro-phenyl)-3,3-dimethyl-ureido]-piperidin-1-yl}-8-aza-bicyclo[3.2.1]octane-8-carboxylic acid ethyl ester
0.0946	2.500	C27H38FN3O3	3-{4-[Cyclohexanecarbonyl-(4-fluoro-phenyl)-amino]-piperidin-1-yl}-8-aza-bicyclo[3.2.1]octane-8-carboxylic acid ethyl ester
0.0985	2.444	C23H35N3O2	3-[4-(ethyl-phenyl-amino)-piperidin-1-yl]-8-azabicyclo[3.2.1]octane-8-carboxylic acid ethyl ester
0.0899	2.552	C21H30FN3O3	4-[Acetyl-(4-fluoro-phenyl)-amino]-[1,4′]bipiperidinyl-1′-carboxylic acid ethyl ester
**Patent title: Muscarinic agonists (US Patent 7378447)**			
0.0985	2.618	C19H29N3O4	Carbamic acid tert-butyl ester (R)-(6-(1-(morpholin-4-yl)ethylideneamino)-2(R)-hydroxyindan-1-yl)amide
0.0963	2.431	C22H36BrN3O3	Biphenyl-4-carboxylic acid (R)-(6-(1-(2-methoxyethyl)methylamino)ethylideneamino)-2(R)-hydroxyindan-1-yl)amide
0.0863	2.579	C31H39N3O3	Biphenyl-1-carboxylic acid (R)-(6-(1-(2-pentoxyethyl)methylamino)ethylideneamino)-2(R)-hydroxyindan-1-yl)amide
0.0958	2.622	C31H37N3O3	Biphenyl-4-carboxylic acid (R)-(6-(1-(2-tert-butoxyethyl)methylamino)ethylideneamino)-2(R)-hydroxyindan-1-yl)amide
**Patent title: Modulators of muscarinic receptors (US Patent Application 20100311746A1)**			
0.0758–0.0985	2.390–2.638		1041 compounds

## Discussion

Brain oxidative stress/status defines the state of sympathoexcitation (increased level of ROS) or sympathoinhibition (the effect of NO), via angiotensinergic neurons (Zimmerman, 2011). Since oxidative injury contributes also to the progressive development of NH, targeting oxidative stress may represent one of promising therapeutic strategies for NH treatment.

**Figure 6:**
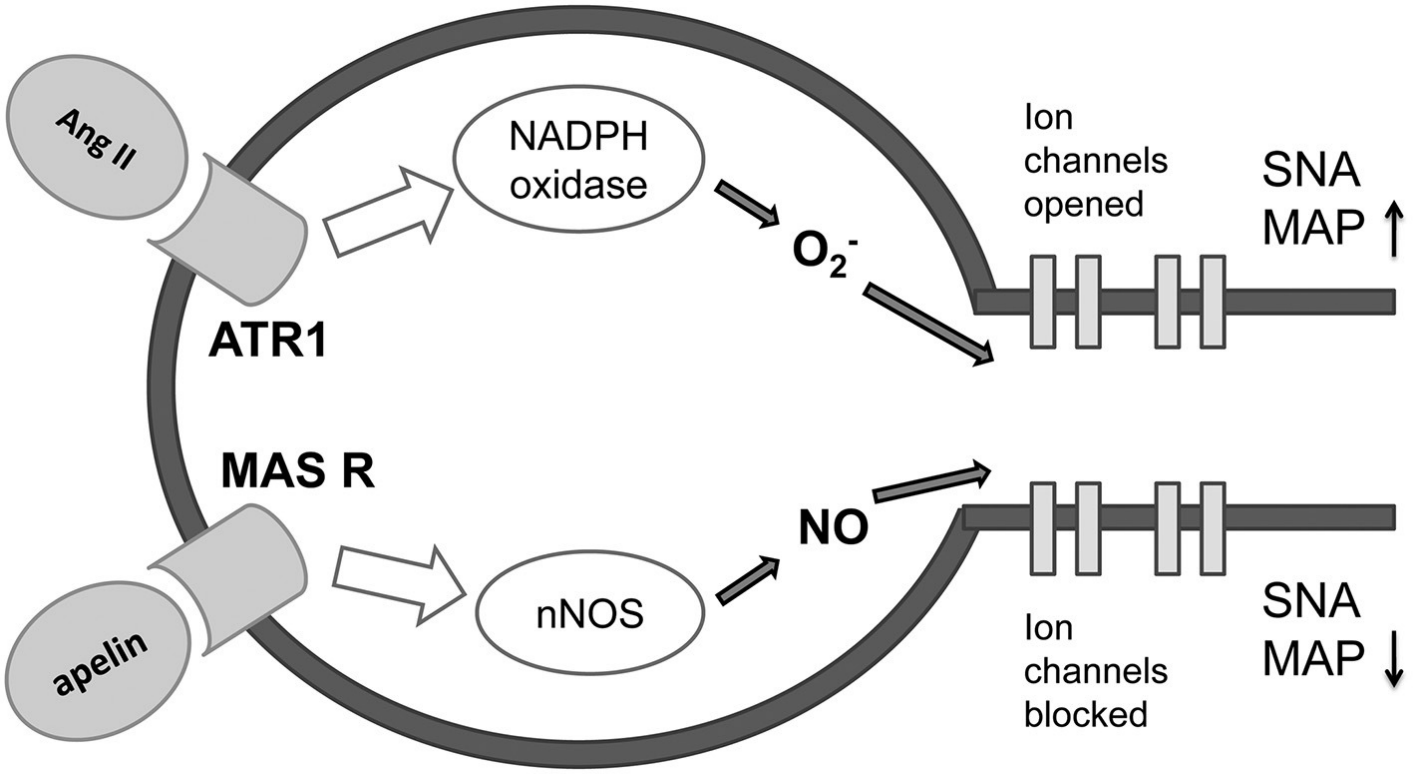
**Proposed mechanism of antagonizing effect of apeline-MAS receptor and Angiotensin II-ATR1 molecular signaling pathways on preganglionic sympathetic neurons in the brainstem (adapted from Northrup and Erickson 1992.)** Ang II, angiotensin II; ATR1, angiotensin 1 receptor; MAS R, MAS receptor; O2, superoxide; NO, nitric oxide; nNOS, neural nitric oxide synthase; SNA, sympathetic nervous activity; MAP, mean arterial pressure.

The ISM analysis identified three forms of apelin as the potential candidates-interactors of MAS receptor on neurons in brainstem sympathetic centers and indicated apelin as potential modulator of MAS1 receptor and novel candidate for treatment of NH (Figure 6). There is lot of recent evidence of the role of apelin as therapeutic agent in protection of the brain against ischemic/reperfusion injury (Yang et al., 2015b), promotion of neurological function recovery after ischemic brain injury (Gu et al., 2013) and potential to cure acute and chronic neurological diseases (Cheng et al., 2012). Synthetic and biased agonists of apelin have been also developed and latter have shown in proof-of-concept studies clinical potential (Yang et al., 2015a). Apelin is adipokine secreted by peripheral tissues but also present in the hypothalamic neurons, having a major impact on the genesis and progression of diabetes (Drougard et al., 2014). This impact is obtained through ROS impact on sympathetic neuronal centers in the diencephalon which cause sympathoexcitation and consequently, the liver glycogenolysis and glusoneogenesis. Our data for the first time point on the potential role of apelin in the development of NH, again, through the action of ROS on sympathetic neuronal centers. Differently from other studies that investigate majorly apelin-APJ receptor signaling pathway (Chun et al., 2008; Yu et al., 2014) our analysis points on the novel mechanism, the apelin-MAS receptor dependent mechanism that could counterbalance sympathostimulating action of ROS. On the basis of the data in the literature (Chun et al., 2008; Yu et al., 2014) and on the basis of our results, there is high probability that apelin has sympathoinhibiting, anti ROS action also on the brainstem level. Further *in vitro* and *in vivo* studies that would elucidate the functional significance of apelin-MAS receptor interaction are needed. This finding could be of importance in designing a novel therapeutics for NH, but also for better understanding and treatment of the states like metabolic syndrome, where diabetes, hypertension, obesity, and obstructive sleep apnea occur in a cluster (Katsiki et al., 2014).

Muscarinic antagonists that were already tested for their efficacy in the treatment of VVS were:

1. atropine(*i.v*.), shown to be fully effective in the cardio-inhibitory form of tilt-induced vasovagal reflex, but with limited action in the vasodepressor form (Santini et al., 1999),
2. propantheline bromide (*p.o*.), shown to be highly effective in preventing VVS. In addition, propantheline bromide's effectiveness is present in the vasodepressor VVS and supports a role of direct cholinergic control of vascular tone (Yu and Sung, 1997),
3. scopolamine (*t.d*.), which showed no effect on VVS (Lee et al., 1996).

An AQVN/EIIP approach for new candidates in pharmacotherapy of VVS reveals that on the basis of their molecular properties, the majority of antimuscarinic drugs (78% of investigated substances) might have therapeutical potential for VVS (Figures 4, 5). More, the remaining 22% were out of AQVN/EIIP active domain, together with scopolamine, the drug that failed to show anti VVS therapeutical effect (Figure 4). Best candidates, identified by AQVN/EIIP approach, for future clinical studies are hydroxysine and cyclopentoate (Figure 5). The suitability of our method was further confirmed by the finding that all muscarinic modulators patented in three patents randomly selected among more than 30,000 patents from the patent database (http://www.freepatentsonline.com) have AQVN/EIIP values within AQVN/EIIP active domain (Table 1, Figure 4).

## Conclusion

Computer-aided design techniques based on the long-range intermolecular interactions offer an insight into therapeutical and pathophysiological aspects of NH and VVS. These bioinformatics approaches also open a promising path for molecular investigation of other NCVD and could be of help in design of *in vitro* and *in vivo* animal and clinical studies of these diseases.

## Author contributions

TB, VP and SG contributed equally to the conception of the work, acquisition, analysis and interpretation of data. TB, VP, and SG participated in drafting the manuscript, revisiting it critically and gave final approval of the version to be published. The authors reached the agreement to be accountable for all the aspects of the work in ensuring that questions related to the accuracy or integrity of any part of the work are appropriately investigated and resolved.

### Conflict of interest statement

The authors declare that the research was conducted in the absence of any commercial or financial relationships that could be construed as a potential conflict of interest.

## Supplementary material

Data Sheet 1 **Sequences of (a) primates MAS1 (b) sequences of primates Angiotensin 1-7 (c) sequences of primates Apelin 28, Apelin 31, and Apelin 36**.

Image 1 **Exported Multiple Sequence Alignment (Edgar, 2004) of MAS1 as artwork using Jalview (Waterhouse et al., 2009)**.
