# Supplementary material for: In silico Therapeutics for Neurogenic Hypertension and Vasovagal Syncope
Source: Front Neurosci. 2016 Jan 21;9:520. doi: 10.3389/fnins.2015.00520 (PMC4720751; doi:10.3389/fnins.2015.00520)
Supplement: Data Sheet 1 — Sequences of (a) primates MAS1 (b) sequences of primates Angiotensin 1-7 (c) sequences of primates Apelin 28, Apelin 31, and Apelin 36. [file DataSheet1.DOC]

Sequences of primates MAS1

>sp|P04201|MAS_HUMAN Proto-oncogene Mas OS=Homo sapiens GN=MAS1 PE=1 SV=1

MDGSNVTSFVVEEPTNISTGRNASVGNAHRQIPIVHWVIMSISPVGFVENGILLWFLCFR

MRRNPFTVYITHLSIADISLLFCIFILSIDYALDYELSSGHYYTIVTLSVTFLFGYNTGL

YLLTAISVERCLSVLYPIWYRCHRPKYQSALVCALLWALSCLVTTMEYVMCIDREEESHS

RNDCRAVIIFIAILSFLVFTPLMLVSSTILVVKIRKNTWASHSSKLYIVIMVTIIIFLIF

AMPMRLLYLLYYEYWSTFGNLHHISLLFSTINSSANPFIYFFVGSSKKKRFKESLKVVLT

RAFKDEMQPRRQKDNCNTVTVETVV

>tr|H2QU00|H2QU00_PANTR Uncharacterized protein OS=Pan troglodytes GN=MAS1 PE=3 SV=1

MDGSNVTSFVVEEPTNISTGRNASVGNTHRQIPIVHWVIMSISPVGFVENGILLWFLCFR

MRRNPFTVYITHLSIADISLLFCIFILSIDYALDYELSSGHYYTIVTLSVTFLFGYNTGL

YLLTAISVERCLSVLYPIWYRCHRPKYQSALVCALLWALSCLVTTMEYVMCIDREEESHS

RSDCRAVIIFIAILSFLVFTPLMLVSSTILVVKIRKNTWASHSSKLYIVIMVTIIIFLIF

AMPMRLLYLLYYEYWSTFGNLHHISLLFSTINSSANPFIYFFVGSSKKKRFKESLKVVLT

RAFKDEMQPRRQEDNCNTVTVETVV

>tr|F7GJU7|F7GJU7_MACMU Uncharacterized protein OS=Macaca mulatta GN=MAS1 PE=3 SV=1

MDGLNVTSSVVEEPTNISTGRNASVGNAHRQIPIVHWVIMSISPVGFVENGILLWFLCFR

MRRNPFTVYITHLSIADISLLFCIFILSIDYALDYELSSGHYYTIVTLSVTFLFGYNTGL

YLLTAISVERCLSVLYPIWYRCHRPKYQSALVCALLWALSCLVTTMEYVMCIDREEESHS

RSDCRAVIIFIAVLSFLVFTPLMLVSSTILVMKIRKNTWASHSSKLYIVIMVTIIIFLIF

AMPMRLLYLLYYEYWSTFGNLHHISLLFSTINSSANPFIYFFVGSSKKKRFKESLKVVLT

RAFKDEMQPRRQEDNCNTVTVETVV

>tr|H2PL76|H2PL76_PONAB Uncharacterized protein OS=Pongo abelii GN=MAS1 PE=3 SV=1

MDGSNVTSFVVEEPTNISTGTNASVGNAHRQIPIVHWVIMSISPVGFVENGILLWFLCFR

MRRNPFTVYITHLSIADISLLFCIFILSIDYALDYELSSGHYYTIVTLSVTFLFGYNTGL

YLLTAISVERCLSVLYPIWYRCHRPKYQSALVCALLWALSCLVTTMEYVMCIDREEESHS

RSDCRAVIIFIAVLSFLVFMPLMLVSSTILVVKIRKNTWASHSSKLYIVIMVTIIIFLIF

AMPMRLLYLLYYEYWSTFGNLHHISLLFSTINSSANPFIYFFVGSSKKKRFKESLKVVLT

RAFKDEMQPRRQEDNCNTVTVETVV

>tr|A0A0D9RJ10|A0A0D9RJ10_CHLSB Uncharacterized protein OS=Chlorocebus sabaeus GN=MAS1 PE=3 SV=1

MDGLNVTSSVVEEPTNISTGRNASVGNAHRQIPIVHWVIMSISPVGFVENGILLWFLCFR

MRRNPFTVYITHLSIADISLLFCIFILSIDYALDYELSSGHYYTIVTLSVTFLFGYNTGL

YLLTAISVERCLSVLYPIWYRCHRPKYQSALVCALLWALSCLVTTMEYVMCIDREEESHS

RSDCRAVIIFIAVLSFLVFTPLMLVSSTILVMKIRKNTWASHSSKLYIVIMVTIIIFLIF

AMPMRLLYLLYYEYWSTFGNLHHISLLFSTINSSANPFIYFFVGSSKKKRFKESLKVVLT

RAFKDEMQPRRQEDNCNTVTVETVV

>tr|G3R4L5|G3R4L5_GORGO Uncharacterized protein OS=Gorilla gorilla gorilla GN=MAS1 PE=3 SV=1

MDGSNLTSFVVEEPTNISTGRNASVGNAHRQIPIVHWVIMSISPVGFVENGILLWFLCFR

MRRNPFTVYITHLSIADISLLFCIFILSIDYALDYELSSGHYYTIVTLSVTFLFGYNTGL

YLLTAISVERCLSVLYPIWYRCHRPKYQSALVCALLWALSCLVTTMEYVMCIDREEESHS

RSDCRAVIIFIAILSFLVFTPLMLVSSTILVVKIRKNTWASHSSKLYIVIMVTILIFLIF

AMPMRLLYLLYYEYWSTFGNLHHISLLFSTINSSANPFIYFFVGSSKKKRFKESLKVVLT

RAFKDEMQPRRQEDNCNTVTVETVV

Sequences of primates Angiotensin 1-7

>sp|P01019|34-40 Angiotensin 1-7 Homo sapiens

DRVYIHP

>tr|H2Q1B7|H2Q1B7_PANTR 34-40 Angiotensin 1-7 Pan troglodytes

DRVYIHP

>tr|G7MFR4|G7MFR4_MACMU 34-40 Angiotensin 1-7 Macaca mulatta

DRVYIHP

>sp|Q9GLP6|34-40 Angiotensin 1-7 Gorilla gorilla gorilla

DRVYIHP

Sequences of primates Apelin 28

>sp|Q9ULZ1|50-77 Homo sapiens

NGPGPWQGGRRKFRRQRPRLSHKGPMPF

>tr|F7GX01|MACMU - Macaca mulatta (Rhesus macaque)F7GX01_MACMU

NGPGPWQGGRRKFRRQRPRLSHKGPMPF

>tr|G3S9L8|GORGO Gorilla gorilla gorilla (Western lowland gorilla)

NGPGPWQGGRRKFRRQRPRLSHKGPMPF

>tr|A0A0D9R7Q2|CHLSB - Chlorocebus sabaeus (Green monkey)

NGPGPWQGGRRKFRRQRPRLSHKGPMPF

Sequences of primates Apelin 31

>sp|Q9ULZ1|47-77 Homo sapiens

GSRNGPGPWQGGRRKFRRQRPRLSHKGPMPF

>tr|F7GX01|MACMU - Macaca mulatta (Rhesus macaque)

GSRNGPGPWQGGRRKFRRQRPRLSHKGPMPF

>tr|G3S9L8|GORGO Gorilla gorilla gorilla (Western lowland gorilla)

GSRNGPGPWQGGRRKFRRQRPRLSHKGPMPF

>tr|A0A0D9R7Q2|CHLSB - Chlorocebus sabaeus (Green monkey)

GSRNGPGPWQGGRRKFRRQRPRLSHKGPMPF

Sequences of primates Apelin 36

>sp|Q9ULZ1|42-77 Homo sapiens

LVQPRGSRNGPGPWQGGRRKFRRQRPRLSHKGPMPF

>tr|F7GX01|MACMU - Macaca mulatta (Rhesus macaque)F7GX01_MACMU

LVQPRGSRNGPGPWQGGRRKFRRQRPRLSHKGPMPF

>tr|G3S9L8|GORGO Gorilla gorilla gorilla (Western lowland gorilla)

LVQPRGSRNGPGPWQGGRRKFRRQRPRLSHKGPMPF

>tr|A0A0D9R7Q2|CHLSB - Chlorocebus sabaeus (Green monkey)

LVQPRGSRNGPGPWQGGRRKFRRQRPRLSHKGPMPF
