# Supplementary figures and images for: In silico Therapeutics for Neurogenic Hypertension and Vasovagal Syncope
Source: Front Neurosci. 2016 Jan 21;9:520. doi: 10.3389/fnins.2015.00520 (PMC4720751; doi:10.3389/fnins.2015.00520)

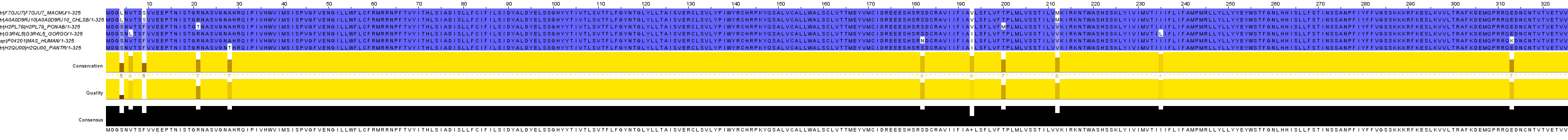

Supplement: Image 1 — Exported Multiple Sequence Alignment (Edgar, 2004) of MAS1 as artwork using Jalview (Waterhouse et al., 2009). [file Image1.JPEG]
